# Neuro-Behcet’s Disease and Its Association With Cerebral Aneurysms and Subarachnoid Hemorrhage: A Case Report

**DOI:** 10.7759/cureus.57275

**Published:** 2024-03-30

**Authors:** Yusra Iqbal, Faatimah Maryam Muzammil, Hana Iqbal

**Affiliations:** 1 Medicine & Surgery, Dubai Medical University, Dubai, ARE; 2 General Practice, Dubai Academic Health Cooperation, Dubai, ARE

**Keywords:** rare diseases, autoimmune vasculitis, neurosurgery, subarachnoid hemorrhage, multiple cerebral aneurysms, neuro-behcet’s disease

## Abstract

Behçet's disease is a rare autoimmune condition characterized by systemic vasculitis, an inflammation of blood vessels, with an unknown etiology. It has varied clinical presentations. Herein, we present the case of a 31-year-old male patient with neuro-Behçet disease who presented with subarachnoid hemorrhage and microaneurysms.

## Introduction

Behcet's disease (BD) is a rare, multisystem inflammatory disorder that predominantly affects individuals aged between 20 and 40. The incidence is higher in males, especially in the high-prevalence areas of Turkey and the Middle East [1].

Vasculitis is a major pathological feature in Behcet's disease, and its pathogenesis remains unknown [2]. The rate of vascular involvement in BD has been reported to vary between 23% and 62%, with arterial involvement being extremely rare, with an incidence of 2.2-7.7% due to vascular neuro Behcet’s disease [3,4]. 

The involvement of the nervous system in BD constitutes the diagnosis of neuro-Behçet’s disease (NBD) [5].

NBD occurs in less than 10% of cases with BD. Individuals who develop NBD typically experience symptoms around 5-6 years after the onset of non-neurological manifestations. Patients may present with motor dysfunction, memory impairment, and personality changes. The symptoms depend on the area of involvement, which may be the spinal cord, brainstem, cerebellum, thalamus, basal ganglia, and internal capsule [6]. Subarachnoid hemorrhage (SAH) and cerebral aneurysms in BD are very rare [7,8]. 

Herein, we report a rare case of neuro-Behçet in a 31-year-old man with a subarachnoid hemorrhage and two tiny blister-bleb aneurysms.

## Case presentation

A 31-year-old male with a history of hypertension and Behçet’s disease presented to the emergency department (ED), complaining of a sudden, severe headache that radiated to the neck and multiple episodes of vomiting.

**Figure 1:**
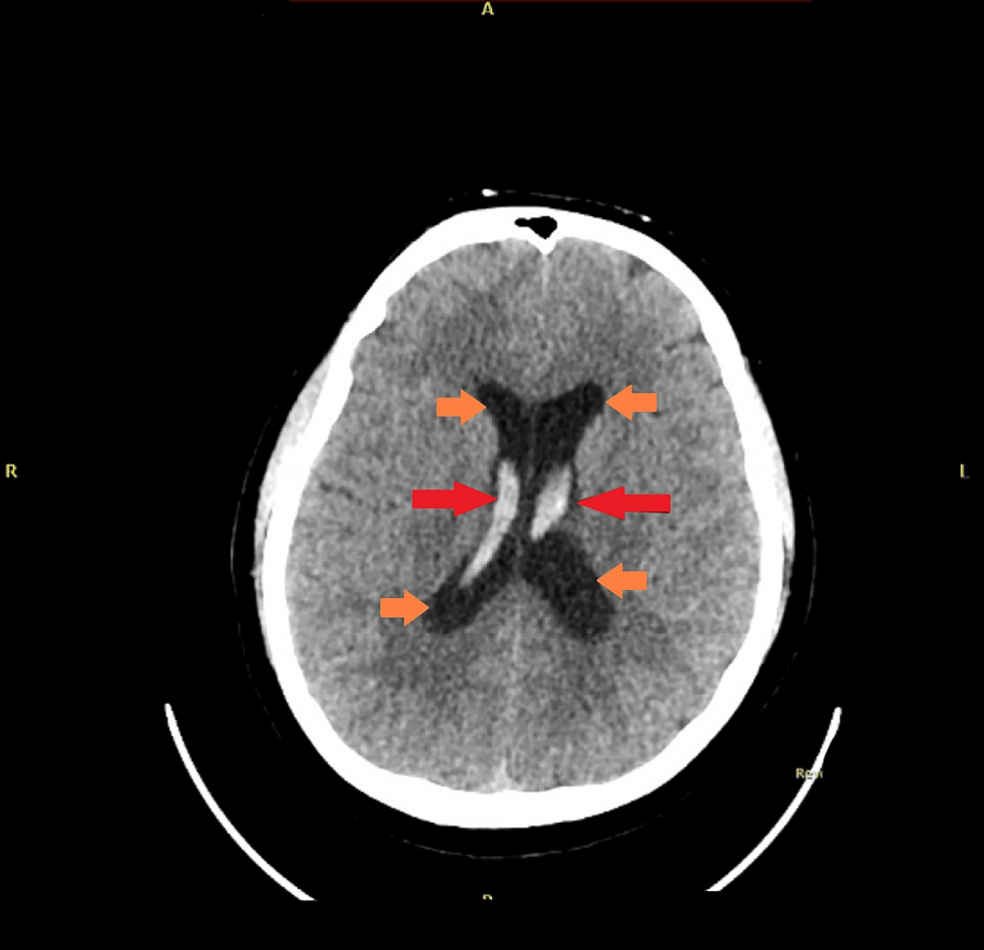
Before external ventricular drain (EVD) placement. There is increased dilatation of the lateral, third, and fourth ventricles (shown by yellow arrows) with mild to moderate intraventricular bleeding (shown by red arrows), with layering noted in the occipital horns. Persistent subarachnoid bleeding is observed in the suprasellar, basal cisterns, and subarachnoid spaces. Mild cerebral edema is noted.

At admission, the patient was conscious, alert, and oriented, with a Glasgow Coma Scale (GCS) score of 15/15. A neurological examination revealed no focal deficits. Cerebral angiography and a CT scan of the brain showed subarachnoid bleeding (Figure 1) and angio-cerebral blister aneurysms located at the right internal carotid artery bifurcation and basilar tip. Investigations done in the ED included the coagulation profile shown in Table 1. 

**Table 1:** Coagulation profile at the time of admission.

Components	Reference range and units	Results at admission
Prothrombin time (PT)	11-14 seconds	14.8 (High)
International normalized ratio (INR)	0.6-1.1	1.19 (High)
Activated partial thromboplastin time (APTT)	28.0-41.0 seconds	34.7

During the stay, he developed dizziness and a near-fainting episode. A repeat CT brain was performed, revealing a new intraventricular hemorrhage (IVH). The patient underwent a burr hole and external ventricular drain placement (EVD). Subsequently, the patient's Glasgow Coma Scale (GCS) declined to 10, and he also developed hydrocephalus. Consequently, the EVD was removed.

A repeat CT brain scan performed showed an interval increase in hydrocephalus. A lumbar puncture (LP) conducted on the same day revealed bloody cerebrospinal fluid (CSF) with an opening pressure of 26 cmH2O and a closing pressure of 6 cmH2O.

A few hours after the lumbar puncture (LP), the patient's condition deteriorated, and the Glasgow Coma Scale (GCS) dropped to 8/15. An urgent CT of the brain revealed a new intraventricular hemorrhage (rebleed). Based on these findings, an urgent right frontal external ventricular drain (EVD) was inserted. The patient was also placed on a ventilator, and the EVD was maintained at 15 cm H2O. After a few days, the EVD was adjusted to 10 cmH2O. The patient was successfully extubated and subsequently transferred out of the intensive care unit (ICU).

**Figure 2:**
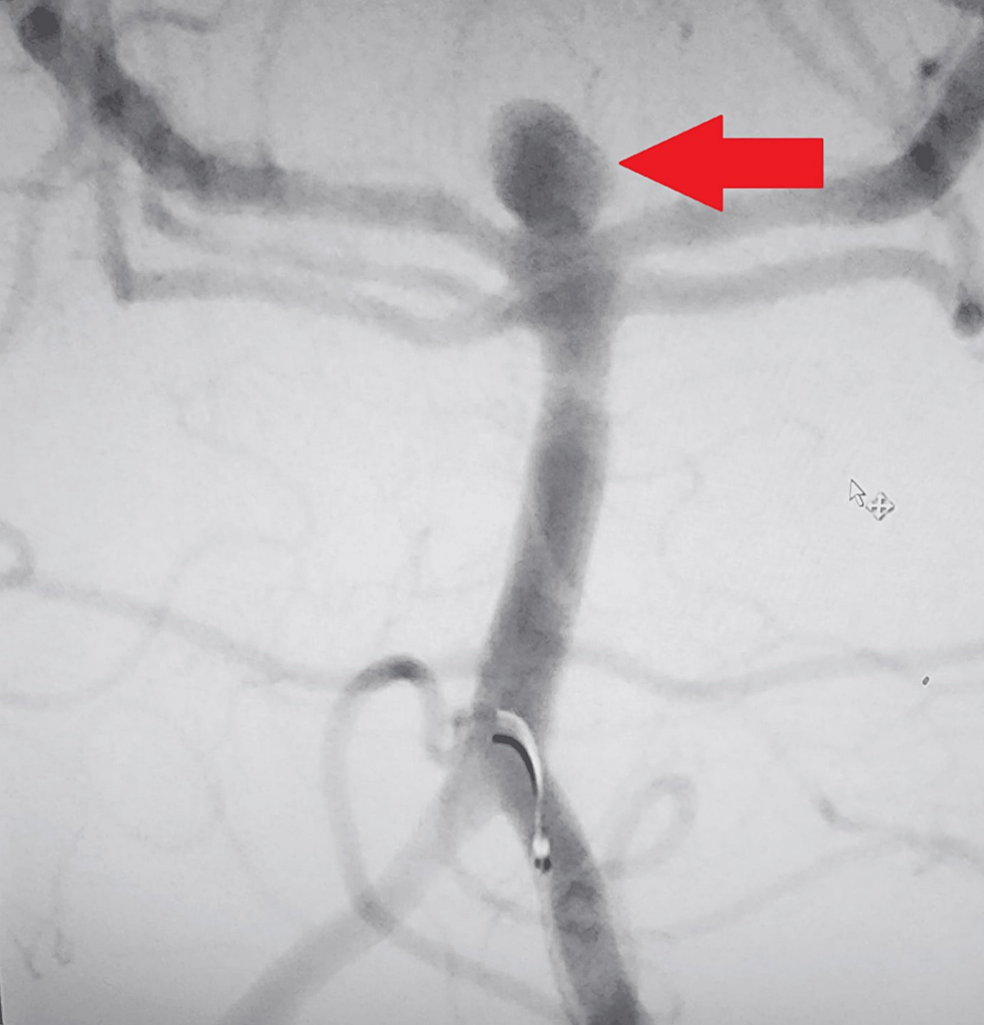
Aneurysm at the tip of the basilar artery, shown by the red arrow.

**Figure 3:**
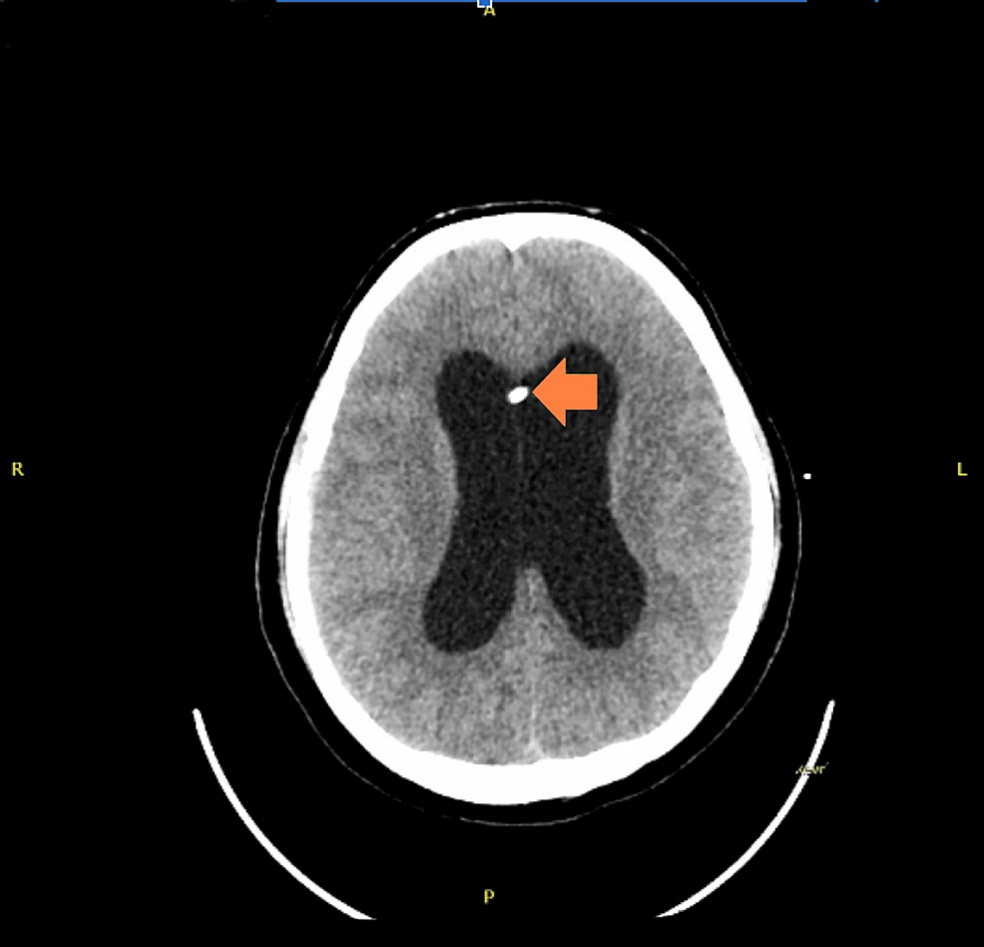
Status post-coiling of the aneurysm at the tip of the basilar artery. A left frontal approach external ventricular drain (EVD) is noted with the tip in the right lateral ventricle (shown by the yellow arrow).

During the hospital stay, he had a positive CSF culture, prompting consultation with the infectious disease team and the initiation of antibiotic treatment. After a few weeks, repeat CT angiography showed evidence of an increasing size of the saccular aneurysm at the tip of the basilar artery (Figure 2). Endovascular management was performed, and he underwent coiling of the basilar artery tip aneurysm, as shown in Figure 3.

Due to his illness and prolonged stay in the hospital, the patient showed signs of depression. A referral to psychiatry was initiated, and after evaluation, the patient was started on Sertraline. Subsequently, his depressive symptoms improved.

The patient received regular physiotherapy for mobilization. He showed gradual improvement and was discharged home with referral appointments for follow-up in multidisciplinary departments, including neurosurgery, psychiatry, and rheumatology.

## Discussion

The initial account of neurological manifestations in BD can be traced to 1941. The term NBD was officially coined by an Italian ophthalmologist [9].

NBD is considered a serious manifestation of BD due to possible complications of severe and permanent neurological deficits, that may negatively impact the quality of life for the patients [10].

Our patient presented with SAH and microaneurysms. The pathophysiology of aneurysms leading to SAH is influenced by two primary factors: the rupture of the internal and external elastic lamina and vasculitis resulting from lymphocytic infiltration of the vasa vasorum [11]. These inflammatory changes may predispose the aneurysm to rebleeding, ultimately leading to SAH in this case. 

The main goal in the treatment of BD is to prevent lasting organ damage by decreasing inflammatory flare-ups and limiting recurrences [9]. Treatment options for cerebral aneurysms in Behçet’s disease depend on factors such as size, location, and whether the aneurysm is ruptured. The use of immunosuppressants and corticosteroids is instrumental in preventing vascular complications [3]. Surgical intervention is the primary choice in cases of ruptured aneurysms. However, the insertion of an arterial catheter itself may pose risks, leading to thrombosis or pseudoaneurysm formation at the puncture site in patients with Behçet’s disease. This may increase the risk of rebleeding or precipitating SAH [11].

In our case, a burr hole procedure with EVD placement was performed. Furthermore, basilar tip aneurysm coiling was also carried out. Corticosteroids and immunosuppressive treatments play a crucial role in preventing vascular complications. Nevertheless, some reports suggest adverse outcomes of steroid therapy in cases of ruptured aneurysm SAH, possibly due to prolonged steroid therapy leading to steroid resistance [1,12].

## Conclusions

In conclusion, we presented the case of a 31-year-old male with a history of hypertension and NBD. The patient's initial complaint of a sudden, severe headache led to the diagnosis of SAH and angio-cerebral blister aneurysms, highlighting the potential severity and complications associated with NBD. The clinical course was marked by a series of critical events, including the development of IVH and hydrocephalus, leading to interventions such as burr hole and EVD placement, followed by a subsequent rebleed and the need for urgent right frontal EVD insertion. This emphasizes the dynamic and unpredictable nature of NBD, necessitating prompt and adaptive management strategies.

Neuro-Behçet’s disease with intracranial aneurysms and SAH is rare but continues to be reported. This case highlights the association of neurovascular complications in Behçet’s disease with inflammatory processes, leading to an increased risk of aneurysm rupture and subsequent SAH. The case also underscores the importance of a multidisciplinary approach, involving neurosurgery, infectious disease, psychiatry, and rheumatology teams, to address the diverse aspects of the patient's condition. The positive outcome achieved through interventions, including the successful management of depressive symptoms and the gradual improvement in physical function through regular physiotherapy, underscores the importance of comprehensive care in NBD. The challenges encountered in this case reinforce the need for ongoing research and awareness regarding the complexities of neuro-Behçet's disease.
